# Development of research on HIV-associated neurocognitive disorder and emerging trends: a visualization analysis via CiteSpace

**DOI:** 10.3389/fimmu.2025.1478187

**Published:** 2025-02-03

**Authors:** Tingting Zhou, Xuannan Chen, Yu Lai

**Affiliations:** ^1^ School of Clinical Medicine, Chengdu University of Traditional Chinese Medicine, Chengdu, China; ^2^ Acupunture and Tuina School, Chengdu University of Traditional Chinese Medicine, Chengdu, China; ^3^ School of Basic Medicine, Chengdu University of Traditional Chinese Medicine, Chengdu, China

**Keywords:** HIVAIDS, HIV-associated neurocognitive disorder, neurocognitive impairment, CiteSpace, bibliometric analysis, visualized analysis, emerging trends

## Abstract

**Background:**

In the combination antiretroviral therapy era, HIV-associated neurocognitive disorder (HAND) is still widespread among HIV-infected individuals. However, there is no effective treatment for HAND, and the exact pathogenic mechanism of HAND remains unknown. This paper aims to provide a reference for further exploration in the field of HAND research.

**Methods:**

We used CiteSpace software to collect 3057 articles related to HAND in the Web of Science Core Collection for comprehensive analysis. Betweenness centrality, count, and burst values were used as indicators in the visualization analysis, aiming to predict future new directions and cutting-edge trends.

**Results:**

The last decade has been the peak period of HAND research, with the most prominent contributions by authors, countries, and institutions being Grant, Igor (135), the USA (2211), and the University of California System (758), respectively. The most frequently cited article is “HIV-associated neurocognitive disorders persist in the area of potent antiretroviral therapy: CHARTER Study.” The hotspots in this field are “neurocognitive impairment,” “central nervous system,” “cerebrospinal fluid,” “HIV-1 tat,” “SIV,” “inflammation,” “infection,” and “pathogenesis.” The current research direction of HAND is focused on exploring the pathogenic mechanism underlying HIV-associated neurocognitive impairment and potential therapeutic targets.

**Conclusion:**

This study provides a bibliometric visualization of HAND-related literature to gain insight into the development and frontiers of this research field. The study also provides scholars with detailed references and identifies future research directions to better promote the development of this field of research.

## 1 Introduction

Human immunodeficiency virus (HIV) continues to have destructive health effects globally, with 40.4 million [32.9-51.3 million] HIV-related deaths to date and an estimated 39.0 million [33.1-45.7 million] people living with HIV currently (1). Despite great advancements in combination antiretroviral therapy (cART), approximately 1.3 million people were newly infected with HIV in 2022 (1). HIV-associated neurocognitive disorder (HAND) persists, and the proportion of HIV-positive patients impacted by HAND varies from 30% to 50% in the modern cART era (2). Thus, HAND is important in AIDS research.

HAND involves different types of cognitive impairments, including impaired attention span, memory, processing speed, and executive function (3). HAND can be divided into three categories on the basis of the severity of the condition: HIV-associated asymptomatic neurocognitive impairment (NCI), HIV-associated mild NCI, and HIV-associated dementia (HAD) (4). Even though the US Food and Drug Administration and the European Medicines Agency have not specifically approved cART to treat HAND, people who begin cART frequently show notable improvements in their cognitive function (5). Nevertheless, except for the most severe dementia manifestations, the incidence and prevalence of HAND have not decreased, and HAND continues to be prevalent in daily clinical practice (6). Recent experimental data indicated that empirically aggressive antiretroviral therapy might not be as successful as it could be in suppressing HAND (7). In addition, researchers have shown that gp120tg mice with a reduction in α7 nicotinic acetylcholine receptors have less responsive A1 astrocytes, which promotes neuronal and cognitive improvements (8) and provides new ideas for HAND interventions. However, there are still uncertainties about whether HAND can be treated. The presence of HAND indicates that HIV is replicating in immune cells in the brain after entering the central nervous system (CNS) during infection (9). HAND is thought to arise as a result of continuous HIV reproduction, host immunological response and inflammation, oxidative stress, and the direct neurotoxicity of viral proteins (10). Host genetics and their heterogeneity are other aspects that promote susceptibility to HAND and largely explain why the human immune response varies in efficacy (2). Furthermore, drug abuse has been demonstrated to accelerate the progression of HAND (11). However, the precise mechanisms through which different classes of drugs contribute to the development of HAND remain unclear. Nevertheless, it is established that drug abuse leads to an increase in CNS dopamine levels and elevated dopamine levels have been shown to exacerbate infections and inflammation, including in macrophages and microglia (the primary targets of HIV in the brain) (12). Currently available human studies, which primarily include non-Latino Whites, have not mediated differences in neuropathogenesis between racial and ethnic groups. However, existing evidence has been available to demonstrate that key physiopathological mechanisms of HIV-associated NCI may differ by race and ethnic group (10). These findings suggest that race and geographical differences play important roles in HAND field research. As mentioned above, the pathogenesis of HAND is complex and involves factors such as the development of HIV infection in the patient’s body, the interaction between the patient and the environment, and the interaction between the patient and drug use. At present, there is no effective treatment for HAND, and the exact pathogenic mechanism of HAND remains unknown.

CiteSpace is a Java-based software for knowledge domain visualization developed by Professor Chao-mei Chen. CiteSpace simplifies the search for significant papers in the literature of a particular knowledge area so that one can search for salient features in the visualization network intuitively (13). Numerous points of contention, disagreement, and conflicting results can be found in the clinical literature on HAND in HIV (10). To our knowledge, no comprehensive and illuminating bibliometric reports have focused on the remaining problems and new research findings in the HAND literature. Therefore, we visualized the field of HAND research through CiteSpace and conducted in-depth analyses of popular topics and cutting-edge trends in different periods to provide references for future HAND research on complex pathogenic mechanisms and new feasible treatment modalities.

## 2 Methods

### 2.1 Data sources

The Web of Science Core Collection (WOSCC) is an independent global citation database covering a wide range of research types and is the only one to take full advantage of the functionality of CiteSpace. To increase the representativeness and reliability of data, we selected the literature through the Science Citation Index-Expanded version (SCIE) of the WOSCC. The detailed search strategy is displayed in **Table 1**. Using an advanced search, we retrieved documents from 1 January 1990 to 31 December 2023. All eligible literature was exported in a plain text file format with a record of “full record and cited references.”

**Table 1:** Search strategy.

Category	Specific Standard Requirements
Research database	Web of Science Core Collection
Citation indexes	Science Citation Index-Expanded
Searching period	1 January 1990 to 31 December 2023.
Language	“English”
Searching keywords	#1 Topic=(“Neurocognitive Disorder*”) OR Topic=(“Kandinsky Syndrome”) OR Topic=(“Clerambault Syndrome”) OR Topic=(“Organic Mental Disorder*”) OR Topic=(“Mild Neurocognitive Disorder”) OR Topic=(“Mild Neurocognitive Disorders”) OR Topic=(“Nonpsychotic Organic Brain Syndrome”) OR Topic=(“Traumatic Psychoses”) OR Topic=(“Neurocognitive Impairment”) #2 Topic=(“HIV”) OR Topic=(“Human Immunodeficiency Virus*”) OR Topic=(“Human T-Cell Lymphotropic Virus Type III”) OR Topic=(“Human T-Cell Leukemia Virus Type III”) OR Topic=(LAV-HTLV-III) OR Topic=(“Lymphadenopathy-Associated Virus*”) OR Topic=(“Virus*,Lymphadenopathy-Associated”) OR Topic=(“Human T Lymphotropic Virus Type III”) OR Topic=(“AIDS Virus*”) OR Topic=(“Acquired Immun* Deficiency Syndrome Virus”) OR Topic=(HTLV-III) #1 AND #2
Document types	“articles” and “review articles”
Data extraction	Export with full records and cited references in plain text format
Sample size	3057
Research database	Web of Science Core Collection

### 2.2 Data analysis

CiteSpace provides researchers with an intuitive and accurate understanding of the hotspots and evolution of research related to the field through visual analyses (14). CiteSpace version 6.3.R1 was employed. The research horizon is no longer limited to the partial contribution of a specific paper but rather shows the academic impact of a paper on the overall development of the field. The knowledge graph consists mainly of nodes and links. Each node in the graph represents an element to be analyzed. Moreover, links represent co-occurrence or citation relationships. In this case, nodes with a high degree of centrality are turning points or key points in the field. Greater centrality reflects a greater representation of the corresponding research element over a certain period.

## 3 Results

### 3.1 Analysis of annual publication volume

**Figure 1:**
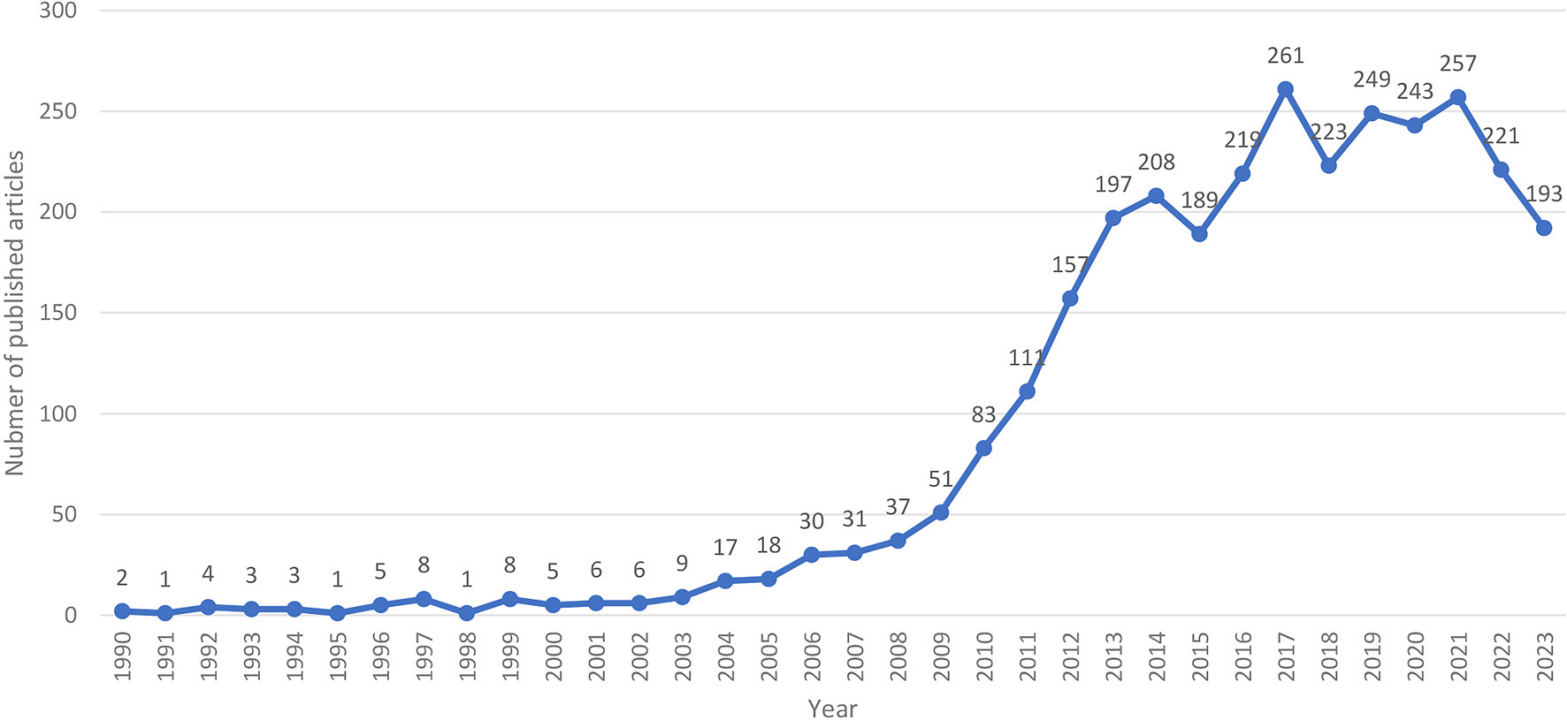
Trend chart of the annual publication volume (1990-2023).

A total of 3057 publications were ultimately included in the analysis, including 2537 articles and 520 reviews. The line graph (**Figure 1**), with the year of publication on the x-axis and the number of papers on the y-axis, visualizes changes in the level of development of a field. Publications based on the topic of HAND have been published every year since 1990. From 1990 to 1999, the number of publications was low, with an average of fewer than 4 publications per year. Since 2004, the number of publications has increased rapidly, peaking in 2017 (261 papers). In the last decade, the number of publications has remained high, with an average of 262 publications per year, indicating that researchers are now paying more attention to HAND.

### 3.2 Analysis of the author cooperation network map

To discover highly influential authors in HAND, we used CiteSpace to generate a distribution map of coauthors with 243 nodes and 358 connections. The size of a circle represents the number of studies published by the author. In the diagram, links between different authors represent collaboration. The thickness of the lines represents the strength of the cooperation, and the thicker the line is, the greater the degree of collaboration between individual authors. **Table 2** lists the top ten authors with the most publications. The top five authors who published a high number of manuscripts were Grant, Igor (159, 5.18%), Ellis, Ronald J (135, 4.42%), Heaten, Robert K (116, 3.79%), Woods, Steven Paul (100, 3.27%), and Letendre, Scott (90, 2.94%).

**Table 2:** The top 10 most productive authors.

Rank	Author	Count of articles	Year of first articles
1	Grant, Igor	159	1997
2	Ellis, Ronald J	135	2006
3	Heaton, Robert K	116	1997
4	Woods, Steven Paul	100	2006
5	Letendre, Scott L	90	2006
6	Moore, David J	88	2011
7	Morgello, Susan	86	2007
8	Letendre, Scott L	85	2006
9	Sacktor, Ned	63	2007
10	Gelman, Benjamin B	60	2010

### 3.3 Analysis of the country and institution cooperation map

**Figure 2:**
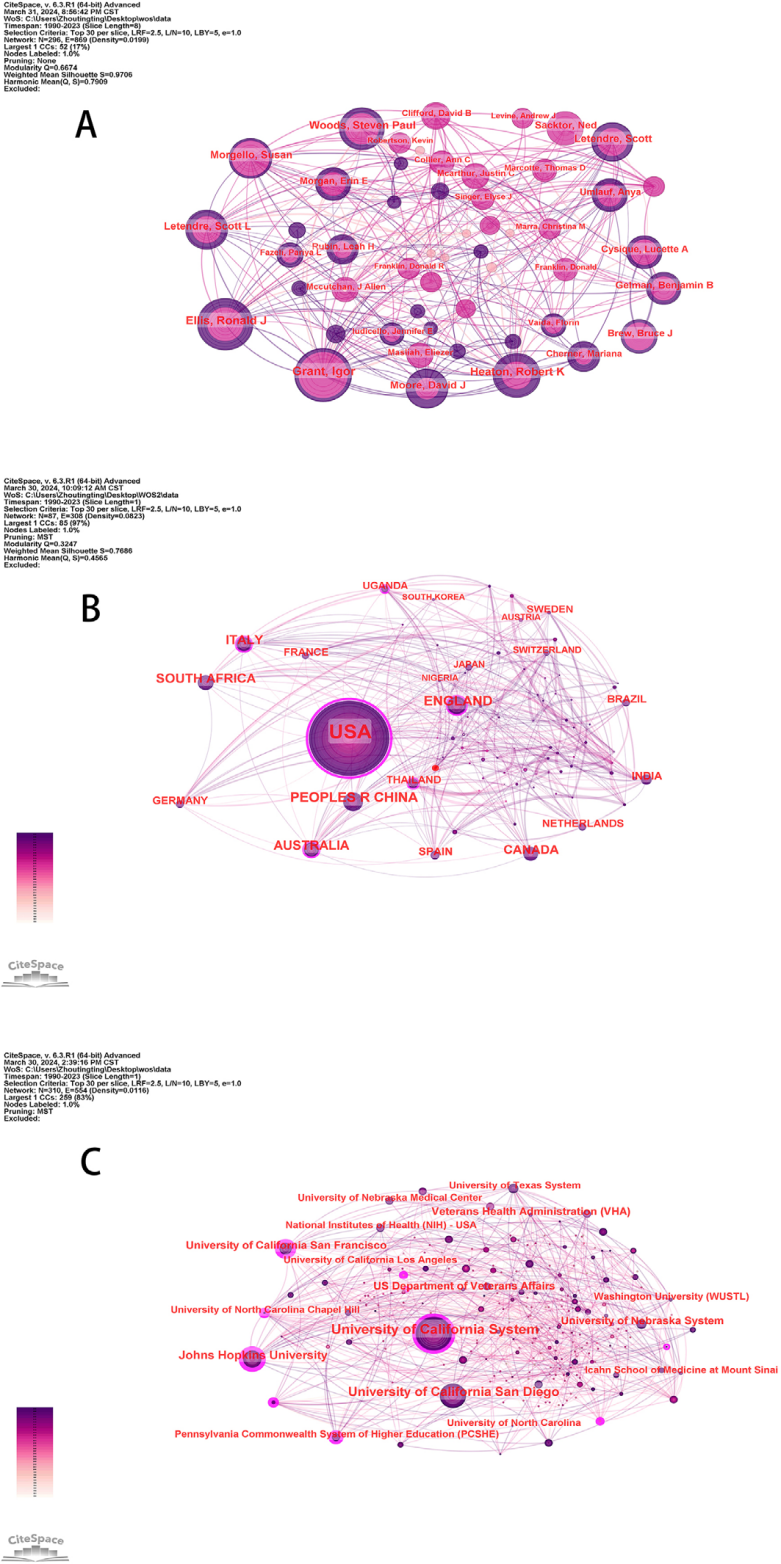
**(A)** The network of coauthors **(B)** The network of countries **(C)** The network of institutions.

After visual analysis with “country” and “institution” nodes, a collaborative map of country and institution networks was produced (**Figures 2B, C**). The purple circle outside the circle represents a betweenness centrality greater than 0.1. Betweenness centrality is used to measure the likelihood of any shortest path through a node in the network graph, which can be used to evaluate the importance of each node in the graph. When the value of betweenness centrality is greater than 0.1, a node is considered critical. **Table 3** shows that the USA has published 2211 (70%) papers, making a significant contribution to HAND research. After the USA, the top five countries are China, England, Australia, and South Africa. Furthermore, we noted that the USA has high country centrality and plays an intermediary role in the national cooperation network. The institution network comprised 310 nodes and 554 links (**Figure 2C**). There are also very strong cooperative links between the various agencies. The 3067 studies were published in 310 institutions; the top five institutions were the University of California System, the University of California San Diego, Johns Hopkins University, the University of California San Francisco, and the University of Nebraska System (**Table 3**). In general, the literature output indicates that there are more universities than hospitals.

**Table 3:** Top ten countries and institutions publishing research on HIV-associated neurocognitive disorder.

Rank	Countries	Centrality	Count	Rank	Institution	Centrality	Count
1	USA	0.60	2211	1	University of California System	0.08	758
2	CHINA	0.08	188	2	University of California San Diego	0.06	519
3	ENGLAND	0.13	167	3	Johns Hopkins University	0.22	272
4	AUSTRALIA	0.15	152	4	University of California San Francisco	0.18	170
5	SOUTH AFRICA	0.10	148	5	University of Nebraska System	0.03	157
6	ITALY	0.10	144	6	US Department of Veterans Affairs	0.04	156
7	CANADA	0.07	120	7	Veterans Health Administration (VHA)	0.02	155
8	SPAIN	0.02	71	8	University of Nebraska Medical Center	0.03	146
9	INDIA	0.06	61	9	University of Texas System	0.04	142
10	GERMANY	0.03	60	10	Icahn School of Medicine at Mount Sinai	0.07	135

### 3.4 Visualized analysis of the dual-map overlay of journals

**Figure 3:**
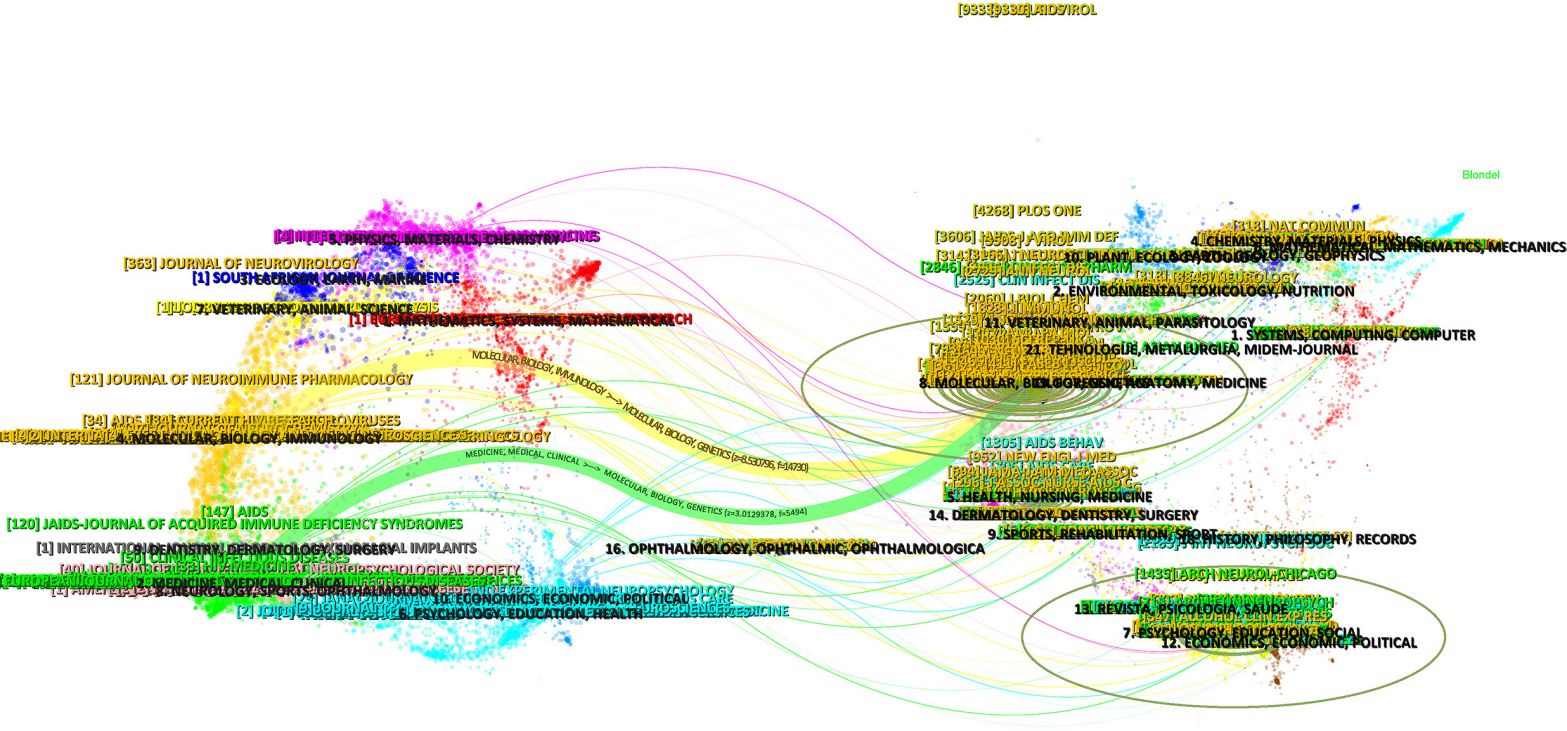
Dual-map overlay of journals that published literature on HIV-associated neurocognitive disorder from 1990 to 2023.

The dual-map overlays of journals allow the visualization of the position of the research topic within the discipline and thus capture the flow of information at the journal level; furthermore, they reflect the dynamics of research on the topic. A dual-map overlay consists of two main parts, with the citing journals on the left and the cited journals on the right. The curve between them is the citation route (15). The dual-map overlay of journals is shown in **Figure 3**. There are two main citation paths, which show that journals published in the fields of molecular/biology/genetics usually influenced journals published in the fields of molecular/biology/immunology and medicine/medical/clinical. This map revealed the cross-citation relationships between journals spanning various disciplines, reflecting the cross-collaboration and dissemination of knowledge within the research field.

### 3.5 Document analysis

#### 3.5.1 Cocited literature analysis

**Figure 4:**
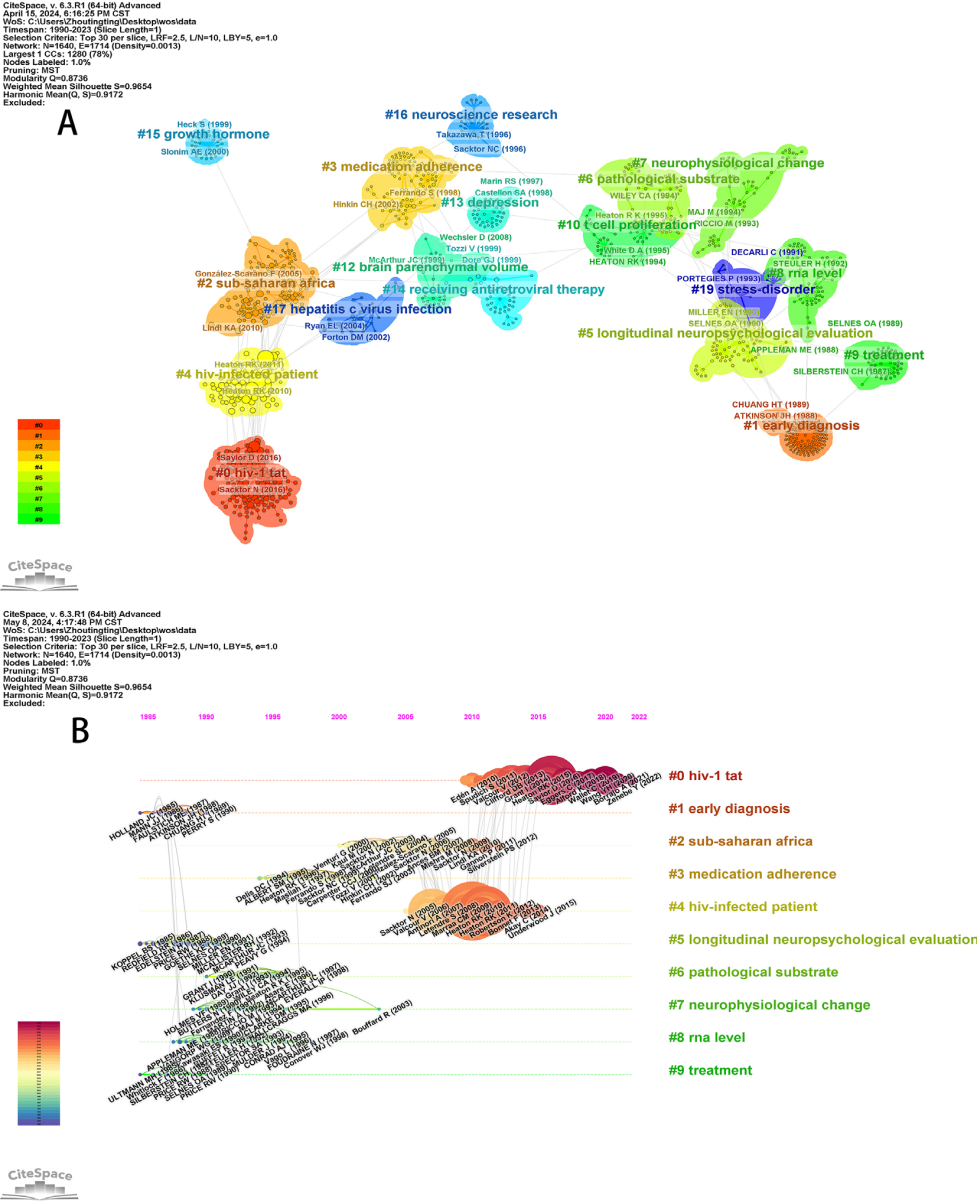
**(A)** Cluster network map of cocited literature **(B)** Timeline map of cocited literature.

The visualization map generated with “reference” as the node includes 1640 nodes and 1714 links (**Figure 4A**). After clustering, twenty groups of cluster labels (#0-19) were generated via the log-likelihood ratio (LLR). CiteSpace employs modularity (Q) and the mean silhouette (S) as the indexes for evaluating the quality of the clusters, which usually take values in the interval [0,1]. Generally, when the Q values are greater than 0.3, clusters develop a significant community structure. When the S value is greater than 0.7, the cluster is efficient and convincing (16). The weighted mean silhouette of this map is 0.9654, indicating that the research field is concentrated and that the overall cluster results are quite good. The maximum and the most recent cluster label (#0), HIV-1 tat, is the HIV-1 transactivator of the transcription protein. Tat is found in the cerebrospinal fluid of HIV patients who are receiving cART, and its expression in animals can cause cognitive symptoms (17). Owing to the role of neuroinflammation, neuronal damage, and NCI in people living with HIV, extensive research has been conducted in clinical and basic research settings, and the latest research has progressed to evaluating tat-induced neuroinflammation (18). In other words, HIV-1 tat provides a new and promising possible avenue for the treatment of HAND.

#### 3.5.2 Cocited literature timeline map

On the basis of the cocited literature clustering map, the ten largest labels are selected to generate the timeline map (**Figure 4B**). The map can reflect the temporal characteristics and evolution trends of the cited literature (16). Notably, cluster label (#0), HIV-1 tat, has been active since 2010 and is still a research hotspot in this field.

#### 3.5.3 Cocited representative literature

The cocitation frequency indicates the degree of the contribution of the literature to related fields (19), and the top ten most representative studies are determined with the cocited frequency as the index (**Table 4**). Three of the top 10 articles are from Robert K. Heaton and his team, ranking first, second, and seventh, respectively, which shows that this team has made outstanding contributions to the field of HAND research. Among these articles, four articles are published in the journal Neurology, indicating the important position of this journal in the field of HAND research.

**Table 4:** The top 10 cocited representative studies on HIV-associated neurocognitive disorder.

Rank	Cited number	Title	Journal	Reference
1	349	HIV-associated neurocognitive disorders persist in the area of potent antiretroviral therapy: CHARTER Study	Neurology	(20)
2	267	HIV-associated neurocognitive disorders before and during the era of combination antiretroviral therapy: differences in rates, nature, and predictors	Journal of NeuroVirology	(21)
3	248	HIV-associated neurocognitive disorder — pathogenesis and prospects for treatment	Nature Reviews Neurology	(22)
4	208	Updated research nosology for HIV-associated neurocognitive disorders	Neurology	(4)
5	137	Cognitive dysfunction in HIV patients despite long-standing suppression of viremia	AIDS	(24)
6	128	Prevalence of HIV-associated neurocognitive disorders in the Multicenter AIDS Cohort Study	Neurology	(31)
7	116	Neurocognitive change in the area of HIV combination antiretroviral therapy: A longitudinal CHARTER Study	Clinical Infectious Diseases	(32)
8	116	Validation of the CNS Penetration-Effectiveness Rank for Quantifying Antiretroviral Penetration Into the Central Nervous System	Archives of Neurology	(33)
9	100	Asymptomatic HIV-associated neurocognitive impairment increases risk for symptomatic decline	Neurology	(34)
10	100	Human Immunodeficiency Virus-Associated Neurocognitive Disorders Mind the Gap	Annals of Neurology	(35)

### 3.6 Keyword analysis

#### 3.6.1 Keyword co-occurrence analysis

**Figure 5:**
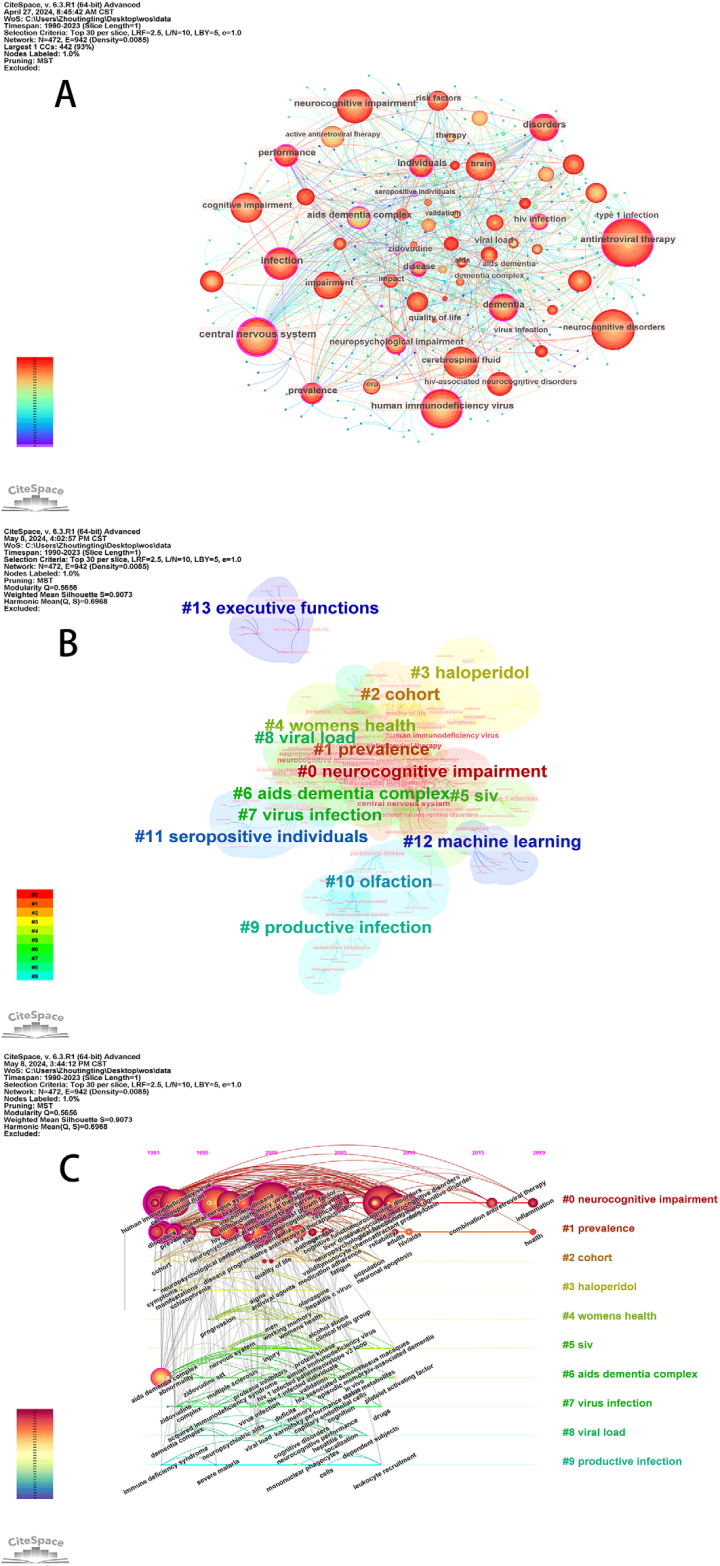
**(A)** Network map of keywords **(B)** Keyword cluster analysis co-occurrence map **(C)** Timeline view of the keyword co-occurrence map.

Nodes represent keywords, and the size of the circles indicates how often these keywords are referenced (**Figure 5A**). The top ten most influential keywords were obtained on the basis of citation frequency (**Table 5**). Highly influential keywords represent popular topics in a field, and nodes marked with purple circles represent excellent betweenness centrality. Therefore, the top ten keywords with high betweenness centrality have unique and important significance in this field. The following is a simple analysis of the top ten keywords with high intermediate centrality. Human immunodeficiency virus (615 times) and the central nervous system (519 times) are the subjects of the study. Antiretroviral therapy (779 times) is the commonly used treatment for HIV patients in modern society. Neurocognitive impairment (442 times) and dementia (335 times) are the two types of HAND classified according to severity. In addition to this basic information, the keywords infection (464 times) and cerebrospinal fluid (440 times) are high-frequency, high intermediary centrality keywords that represent the primary research direction of the field.

**Table 5:** The top 10 representative keywords on HIV-associated neurocognitive disorder.

Rank	Keywords	Count	Centrality
1	antiretroviral therapy	779	0.13
2	human immunodeficiency virus	615	0.11
3	neurocognitive disorders	574	0.03
4	central nervous system	519	0.21
5	infection	464	0.15
6	neurocognitive impairment	442	0.1
7	cerebrospinal fluid	440	0.1
8	cognitive impairment	423	0.04
9	impairment	367	0.08
10	dementia	335	0.13

#### 3.6.2 Keyword cluster and timeline map analysis

Cluster analysis of keywords for better representation of the research frontiers of a particular topic is performed by using a spectral clustering algorithm to cluster the keywords, and the LLR algorithm is used to extract the keywords from the cited articles to annotate the clusters. It is generally accepted that when the Q value is greater than 0.3, the formation of community structures is significant; when the S value is greater than 0.7, clustering is efficient and convincing (16). Finally, 14 clusters were retained, with a Q value of 0.5656 and an S value of 0.9073 (**Figure 5B**), indicating that the results are credible and valuable for research. The high degree of overlap of the clustered color blocks indicates that the different parts are closely related and interact with each other. The keyword timeline view shows the dynamic evolution path of the research hotspots represented by the keywords. **Figure 5C** clearly displays the stage hotspots and development directions of HAND research from the dimension of time. The top 10 clusters were retained in the timeline graphs and included NCI, prevalence, cohort, haloperidol, health, SIV, AIDS dementia complex, virus infection, viral load, and productive infection. Currently, cluster #0 (“neurocognitive impairment”) and cluster #1 (“prevalence”) are still active, with new keyword generation. The debate that has arisen in recent years over the diagnostic criteria for HAND is a key reason why HAND prevalence is a popular topic of discussion. NCI is the most common and prevalent symptom of HAND under combination antiretroviral therapy. Studies on NCI in HAND patients have remained a popular research topic from 1990 to the present. Recent explorations of CNS inflammation may provide new opportunities to identify treatments for NCI in HAND patients. In addition, although SIV no longer appears as a relevant keyword in the timeline graph in recent years, there is a strong link between SIV and the high-frequency keyword phrases that are still active in the clustering graph, which may suggest that the current research direction of HAND is still inseparable from SIV.

#### 3.6.3 Keywords with citation bursts

**Figure 6:**
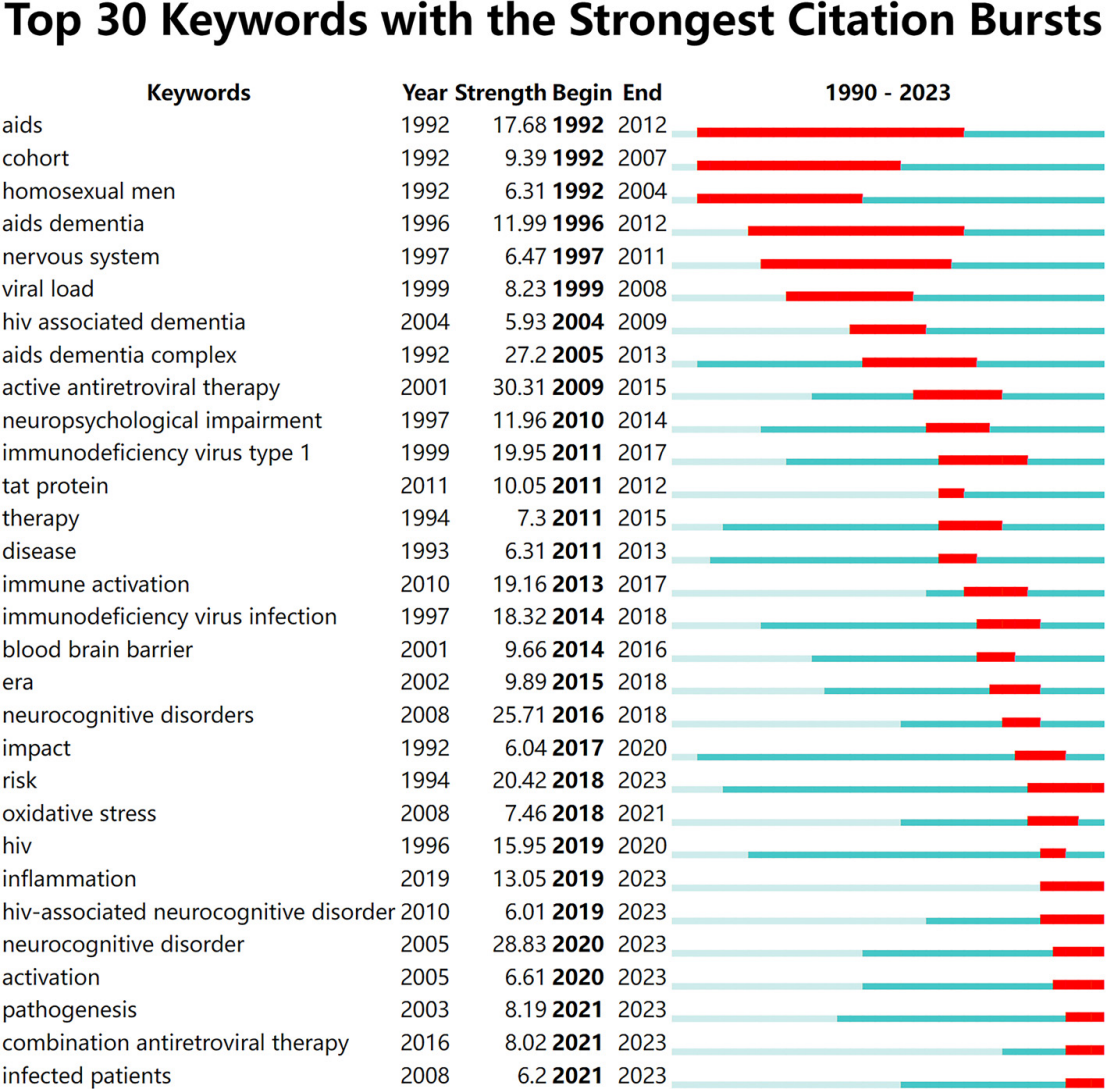
The top 30 keywords with the strongest citation bursts.

Burst word detection algorithms explore cutting-edge trends in research areas on the basis of the rate of increase in the frequency of keyword occurrences. The red line indicates the period of the keyword burst, and the blue line indicates the time interval. The popular frontiers of HAND in different years are analyzed on the basis of the time and duration of keyword bursts. **Figure 6** shows the situation of the top 30 keywords with the strongest citation bursts. The top five keywords in terms of explosive intensity are active antiretroviral therapy (30.31), neurocognitive disorder (28.83), AIDS dementia complex (27.2), neurocognitive disorders (25.71), and risk (20.42). On the basis of the starting year of keyword appearances in the map (**Figure 6**), the exploration of HAND has been a gradual process, with different keywords appearing from 1992 to 2016. The most recent keyword citation peak occurred in 2021 (pathogenesis, combination antiretroviral therapy, infected patients). Notably, “risk,” “inflammation,” “HIV-associated neurocognitive disorder,” “neurocognitive disorder,” “activation,” “pathogenesis,” “combination antiretroviral therapy,” and “infected patients” are still showing ongoing bursts at present and may indicate the research hotspots of the future.

## 4 Discussion

### 4.1 Current status of research

In this study, 3097 articles related to HAND obtained from the WOSCC were analyzed via bibliometric methods. The basic information, including annual publication quantity, author, country, and institution, was analyzed quantitatively. From 1990 to 2023, the number of published articles showed an overall upward trend (**Figure 1**), which can be divided into three stages. The period before 2004 was the initial stage of HAND research, with a few articles published and a slow growth in the number of articles published. The basic research that occurred in this period laid a solid theoretical foundation for the development of HAND research. From 2004 to 2013, the number of articles published increased rapidly, and there was no obvious slowing trend, which indicated that the research had not yet entered the mature stage and that the field of HAND research still had great potential. The last decade has been the peak period of HAND research. According to the visualized analysis of authors (**Table 2**, **Figure 2A**), the most widely published authors in the field of HAND research were Igor Grant, Ronald J Ellis, and Robert Heaton, which indicated that these three authors had made the most outstanding contributions to the field of HAND research. Regarding countries, the USA, China, and England published the greatest number of articles (**Table 3**). In particular, the number of publications and intermediary centralities in the USA was much greater than that in other countries, which indicated that the USA made enormous contributions and played a vital intermediary role in facilitating the integration and exchange of HAND research achievements internationally. The top 10 institutions (**Table 3**) were all from the USA, further illustrating the outstanding contribution of the USA to the field of HAND research. The University of California System has the highest number of published articles, whereas Johns Hopkins University has the highest number of intermediary centralities. Cooperation with countries that are more specialized in a specific research field can promote other countries’ understanding of that field and thus better promote the development of that research field. Therefore, we advocate in-depth research cooperation and communication between different countries and institutions to eliminate academic barriers and promote the development of the HAND research field. **Figure 3** shows that papers published in MOLECULAR, BIOLOGY, and GENETICS journals were frequently cited in papers published in MOLECULAR, BIOLOGY, and IMMUNOLOGY journals and MEDICINE, MEDICAL, and CLINICAL journals. This means that current trends in HAND research focus on transition medicine and basic research.

### 4.2 Important research findings

According to the citation frequency analysis of the literature, the paper published in the journal “Neurology” by Robert K. Heaton (20) had the most citations. The article, presenting the findings of a cross-sectional, observational study, clearly states that the most severe HAND diagnosis (HAD) was rare, but milder forms of impairment remained common in the era of potent antiretroviral therapy. Moreover, future studies could clarify whether early disease events may trigger chronic CNS changes and whether early cART prevents or reverses these changes. The second most frequently cited article was published in the “Journal of NeuroVirology” by Robert K. Heaton (21), who used comparable methods of subject screening and assessments to classify neurological deficits in HIV-positive and HIV-negative participants in the pre-cART era and the cART era to demonstrate that a high prevalence of HIV-associated NCI persists at all stages of HIV infection and suggested that early treatment to prevent severe immunosuppression may help to prevent HAND. The third most frequently cited article, published in the journal “Nature Reviews Neurology” by Justin C. McArthur (22), was a review of the epidemiology of HAND, the evolving concepts of its neuropathogenesis, novel insights from animal models, and new approaches to treatment. This article also discusses how inflammation is sustained in chronic HIV infection and suggests adjunctive therapies—treatments targeting CNS inflammation and other metabolic processes. The fourth most cited article was published in 2007 by Justin C. McArthur (4), who proposed explicit algorithms to aid in the standardized diagnostic classification of HAND. Although this set of classification criteria has been widely used for many years, it has been suggested that the current approach to categorizing cognitive impairment in people living with HIV may overestimate the burden of the disease and, in turn, affect the emergence of uncertainty in the exploration of disease mechanisms (23). Therefore, a new diagnosis and categorization of cognitive disorders in HIV-infected patients is currently being discussed at the forefront of research, which is a landmark in the field of cognitive disorders in HIV-infected patients. The fifth most cited article was authored by Renaud A. Du Pasquier (24), whose research demonstrated a significant prevalence of HIV-associated NCI and identified it as the most common subtype of HAND in HIV-positive patients with chronic clinical deficiency viremia, as assessed by a scale with a cutoff threshold of 14 points or below. A discussion of the top five highly cited articles and their specific publication years showed that the prevalence of the most severe subtype of HAD declined in the cART era and that the prevalence of HIV-associated NCI remained high in the clinical setting between 2004 and 2014. The specific pathogenesis and ways to reduce the prevalence of HIV-associated NCI have also become important directions of HIV research in the last decade.

### 4.3 Research hotspots and frontiers

The frequency and centrality of keywords show that the current international research hotspots for HAND are the CNS, infection, NCI, and cerebrospinal fluid. Analysis of the keywords and references in the clusters reveals that the dominant focus areas of HAND research are HIV-1 tat, SIV, neurocognitive impairment, and prevalence. According to the burst detection analysis, the top five burst keywords are active antiretroviral therapy, neurocognitive disorder, AIDS dementia complex, neurocognitive disorders, and risk. Among the emerging trends and research frontiers of the current research topics are inflammation, activation, pathogenesis, combination antiretroviral therapy, and infection.

#### 4.3.1 HAND research directions

From the combined analysis of the keywords, it is clear that the type of HAND has progressed from “AIDS dementia complex” (2005-2013) to “neurocognitive disorders” (2016-2018) to “neurocognitive disorders” (2020-2023) in the cART era, which means that the current research focus and direction are HIV-associated NCI, which is consistent with the results of the visual analysis of the literature.

#### 4.3.2 HAND pathogenesis and therapeutic directions

In clustering and timeline analyses of the related literature, one of the most important research points to date has been HIV-1 tat, which continues to be produced even after HIV replication has been suppressed by antiretroviral therapy and is toxic to neurons and glia (18). Recent studies have proposed that HIV-1 viral proteins are induced in neuroinflammation through specific pathways, including multiple regulatory protease pathways. In addition, changes in tat sequence can affect inflammation and the resulting neuronal consequences (18). Inflammation is another important keyword in the field of HAND research. Tat-induced neuroinflammation has also been a major area of exploration in basic research. SIV is one of several strains of retroviruses of the genus Lentivirus that cause AIDS-like immunodeficiency syndrome in primates by infecting helper T cells of the immune system. New findings in SIV-infected animals have demonstrated that intestinal damage may lead to the activation of brain cells, suggesting that intestinal damage may contribute to brain damage during SIV and HIV infection (25). Furthermore, studies in SIV-infected animals have shown that long-term low-dose delta-9-tetrahydrocannabinol (THC), which can lead to the production of neuroprotective metabolites, may reduce neuroinflammation by increasing endogenous cannabinoid levels and promoting the growth of intestinal bacteria to positively regulate the microbiota-gut-brain axis (MGBA) (26). And by revealing the immunological and pathological effects of different myeloid phenotypes in the acutely SIV-infected brain, the study suggests a complex interplay between viral infection, the innate immune response, and the central nervous system, which provides valuable insights into the search for possible targets for HAND (27). More importantly, the inability of cART to suppress the virus may be ameliorated. Not only may People living with HIV on cART benefit from studies through the SIV animal series, but also those patients who do not have access to cART. Thus, SIV has also become an important area in the field of HAND research to find effective treatments. Research on the topic of HAND is conducted against the backdrop of the complexity of its pathogenesis and a lack of therapeutic approaches, with lesions in the CNS being among the most direct and definitive manifestations. Investigating the specific pathogenic mechanisms underlying HIV-associated NCI by observing changes in substances in the cerebrospinal fluid is a commonly used research approach. In a recent study, researchers examined cerebrospinal fluid, serum, and plasma levels of neurofilament light and tau and concluded that increased levels of these proteins are associated with poorer neurocognitive performance (28). Cerebrospinal fluid substance change assays are valuable for studying the specific pathogenesis of HAND. Meanwhile, brain cell-derived extracellular vesicles are a novel means of studying valuable molecular information in HAND that can compensate for existing expensive neuroimaging techniques and relatively invasive measures. Despite the current lack of specificity of extracellular vesicles (EVs) markers in the CNS, rapid developments in the field of EVs will continue to guarantee a more reliable way of studying HAND (29). Furthermore, recent studies have shown that astrocyte-derived extracellular vesicle (ADEV) carrying amyloid cargo plays a role in mediating synaptic degeneration associated with HAND and that HIF-1α protects against the damaging effects of HIV-1 Tat-ADEV (30). These findings will advance neuropathological research on HIV-associated neurocognitive disorders. In addition, promising targets for research into the treatment of NCI in HIV-infected patients include human growth hormone-releasing hormone (hGHRH) and the enzyme phosphatidylinositol-glycan-specific phospholipase D (also known as GPLD1) (10), and even though there are difficulties in the complete treatment of HAND, multidimensional exploration for treatments never ceases.

### 4.4 Limitations

The study has the following limitations: first, articles were obtained only from WOSCC, and the data analyzed were not comprehensive; second, there is a constant stream of data updates in the field of study, and as HAND is explored in depth in the future, bibliometric analyses may differ from actual studies.

## 5 Conclusions

With the increasing use of cART, the AIDS dementia complex, the most severe form of HAND, has become clinically rare, whereas HIV-associated NCI remains common and has become the most critical area of research on the topic of HAND in the last decade. Because of the complexity of HAND pathogenesis and the diversity of its clinical manifestations, no accurate and effective treatment has yet been identified. Through CiteSpace statistics, we found that HIV-1 infection and induced inflammation are essential indicators for identifying the specific pathogenesis of HAND. Moreover, research on SIV animal models can provide more possibilities for research on HAND. Furthermore, the combined factors of the presence of virus in the cerebrospinal fluid of HIV patients, persistent inflammation, and the direct or indirect neurotoxicity of antiretroviral drugs cannot be ignored. Therefore, more research is needed to determine the specific pathogenesis of HAND and its clear therapeutic targets and effective treatments.

## Data Availability

The raw data supporting the conclusions of this article will be made available by the authors, without undue reservation.
